# Clinical Audit on Venous Thromboembolism (VTE) Risk Assessment and Guideline Adherence in Surgical Practice

**DOI:** 10.7759/cureus.93708

**Published:** 2025-10-02

**Authors:** Mohand Tag Elsser Mohammed Albadwy, Abubaker Osman Mohammed Ibrahim, Bishoy Alfred William Dawis, Mohammed Osman Ahmed Osman, Ali Shamsaldeen, Shima Abdalraheem Elnadeef Hussein, Sahar Moudathir Yousif, Mogahid Hamdan Adam Ahmed, Saria Abdulgadir, Ahmed Ibrahim Hamed Mohamed, Romisaa Elamin, Mohamed Eltayeb Elnour, Ahmed Alhaj, Mohamed Abd Alkariem Ahmed Ibrahim, Ahmed Mohamed, Seddig Alkazem Mohammed Alkheir, Amany Amin Ali Hussien, Fatima Eltahir, Mustafa Mohamed, Basil M Mohamed

**Affiliations:** 1 Surgery, National University - Sudan, Khartoum, SDN; 2 Uorology, Atbara Police Hospital, Atbara, SDN; 3 General Surgery, Atbara Teaching Hospital, Atbara, SDN; 4 Internal Medicine, The National Ribat University, Khartoum, SDN; 5 General Surgery, Sudan Medical Specialization Board, Khartoum, SDN; 6 Trauma and Orthopaedics, Rotherham General Hospital, Rotherham, GBR; 7 General Surgery, Prince Osman Digna Referral Hospital, Port Sudan, SDN; 8 General Surgery, University of Kordofan, El-Obeid, SDN; 9 Orthopedics and Trauma, Saudi German Hospital, Dammam, SAU; 10 General Surgery, Prince Osman Digna Teaching Hospital, Port Sudan, SDN; 11 Medicine, Atbara Police Hospital, Atbara, SDN; 12 Trauma and Orthopaedics, Our Lady of Lourdes Hospital, Drogheda, IRL; 13 Orthopedic Surgery, Sudan Medical Specialization Board, Khartoum, SDN; 14 Orthopaedics and Trauma, Gezira Centre for Orthopaedic Surgery and Traumatology, Wad Madani, SDN; 15 General Surgery, Atbara Teaching Hospital, Khartoum , SDN; 16 Pediatric Surgery, Sudan Medical Specialization Board, Khartoum, SDN; 17 Internal Medicine, Prince Osman Digna Teaching Hospital, Port Sudan, SDN; 18 Anatomy, National University - Sudan, Khartoum, SDN

**Keywords:** caprini score, clinical audit, guideline adherence, patient education, quality improvement, risk assessment, surgical inpatients, thromboprophylaxis, venous thromboembolism, vte prophylaxis

## Abstract

Background: Venous thromboembolism (VTE) is a leading cause of preventable complications among surgical inpatients, yet compliance with risk assessment and prophylaxis often falls short, particularly in resource-limited settings. This audit aimed to evaluate and enhance VTE prevention practices through structured interventions.

Methods: A closed-loop audit was performed in two cycles: an initial baseline assessment of 49 surgical inpatients, followed by a re-audit of 53 patients after implementing targeted interventions. Surgical inpatients were assessed using an evidence-based proforma adapted from international guidelines. Interventions included staff education, standardized documentation tools, visual reminders, and electronic prompts. Outcomes focused on the completion of risk assessments, appropriateness of prophylaxis, and delivery of patient education.

Results: Caprini score calculation improved from 45/49 (91.8%) in the first cycle to 53/53 (100%) in the second (p = 0.107). Documentation of prophylaxis duration rose significantly from 22/49 (44.9%) to 48/53 (90.6%, p < 0.001). Guideline-consistent management plans increased from 23/49 (46.9%) to 45/53 (84.9%, p < 0.001). Patient education demonstrated the largest significant improvement, from 14/49 (28.6%) to 43/53 (81.1%, p < 0.001). Doctors’ awareness and adherence to appropriate prophylaxis also improved, with elimination of inappropriate use in low-risk patients and a substantial rise in extended prophylaxis for high-risk cases.

Conclusion: This audit demonstrates that low-cost, multifaceted interventions can strengthen VTE prevention in a resource-limited Sudanese hospital. The unique contribution of this study lies in adapting international guidelines to the Sudanese hospital context, thereby offering a feasible and context-sensitive model for comparable low-resource settings. Sustained improvement will require standardized tools, continuous education, and regular re-auditing.

## Introduction

Blood clots forming in the veins, a condition medically termed venous thromboembolism (VTE), can manifest as deep vein thrombosis (DVT) or travel to the lungs as a pulmonary embolism (PE). Within hospital settings, these events remain a major contributor to preventable deaths, with global estimates suggesting that they account for roughly one in 10 inpatient fatalities [1,2]. Beyond mortality, survivors frequently experience prolonged illness, disability, and reduced quality of life. Recognizing this burden, the United States Surgeon General issued a landmark public health alert in 2008 calling for urgent action against DVT and PE [3]. The report highlighted staggering statistics i.e. hundreds of thousands of Americans develop DVT each year, and more than 100,000 die from PE.

Fortunately, preventive strategies substantially reduce risk [1,4]. These include pharmacological methods, which act by reducing the blood’s tendency to clot, and mechanical approaches, which promote venous flow. When applied appropriately, best-practice prevention can reduce DVT occurrence by more than two-thirds [1]. However, pharmacological agents also carry bleeding risks [5], underscoring the need for individualized risk assessment rather than uniform prescribing [6,7].

Over the past two decades, professional bodies such as the American College of Chest Physicians (ACCP), the National Institute for Health and Care Excellence (NICE), and the American Society of Hematology (ASH) have issued evidence-based guidelines to direct VTE prevention in hospitalized patients [8-10]. These protocols emphasize identifying at-risk patients, initiating tailored prophylaxis, and ensuring timely reassessment throughout hospitalization.

Despite the clarity of these recommendations, consistent application in clinical practice remains suboptimal. Quality improvement studies show that multifaceted interventions, combining education, audit and feedback, and decision-support tools, achieve markedly higher compliance than passive dissemination alone [11]. Even so, mechanical prophylaxis remains unevenly implemented, limiting overall effectiveness and contributing to avoidable morbidity and mortality.

The primary objective of this audit was to evaluate compliance with international standards for VTE prevention at Atbara Teaching Hospital, specifically examining the completeness and timeliness of risk assessments and the appropriateness of prescribed prophylaxis. The secondary objective was to assess doctors’ awareness and practices regarding VTE prevention and to evaluate the impact of targeted interventions introduced between the baseline and re-audit cycles.

## Materials and methods

Study design and setting

This project was conducted as a closed-loop clinical audit with both retrospective and prospective components, aimed at evaluating and improving compliance with VTE risk assessment and prophylaxis among surgical inpatients at Atbara Teaching Hospital, a regional referral center providing a comprehensive range of elective and emergency surgical services. The audit was designed according to internationally recognized VTE prevention guidelines, including those of NICE and ACCP [8,9]. The primary objective was to assess the completeness and timeliness of patient VTE risk assessments and the appropriateness of prescribed prophylaxis, and a secondary objective was to evaluate doctors’ awareness, knowledge, and clinical practices regarding VTE risk assessment and prophylaxis.

Audit cycles

The first audit cycle was conducted retrospectively between October 2024 and January 2025, reviewing patient records to establish baseline adherence. All eligible patient records during this period (n = 49) were included in the analysis. The second cycle was carried out prospectively from March 2025 to July 2025, where all consecutive eligible surgical inpatients were included (n = 53). A consecutive sampling approach was applied in both cycles to ensure inclusion of every eligible case, minimizing the risk of selection bias. Thus, total coverage was achieved in both cycles following a two-month intervention period. All adult surgical inpatients during the audit periods were eligible if they had undergone surgery and had documented VTE risk assessment and prophylaxis. Patients under 12 years of age, day-case surgeries with hospital stays under 24 hours, non-surgical admissions, incomplete or illegible records, and orthopedic trauma cases were excluded.

Data collection

Data were collected using a standardized audit proforma adapted from international guidelines, modified to suit local clinical practice and available resources. The tool captured key parameters, including completion of VTE risk assessment at admission, reassessment within 24 hours post-surgery, type and appropriateness of prophylaxis, and documentation of patient-specific risk factors. A pilot review of 10 records was undertaken to refine the tool and ensure inter-rater reliability. Data were extracted from medical charts, operative notes, prescription records, and hospital logs and entered into Google Forms and Microsoft Excel for analysis. Ambiguous entries were resolved by consensus among the audit team, and missing or unclear data were coded as “non-compliant” to maintain completeness.

In parallel, the audit assessed doctors’ awareness and clinical practices regarding VTE prophylaxis. All medical officers, registrars, and house officers involved in the care of surgical patients during the audit periods were invited to participate. A structured questionnaire was developed to evaluate clinicians’ knowledge and practice. The questionnaire addressed the primary method of VTE risk assessment, approaches to risk stratification, frequency of using Caprini score parameters, use of pharmacological prophylaxis for low- and high-risk patients, and provision of patient education. Responses were collected retrospectively during the first cycle and prospectively in the second cycle after the intervention phase, and all responses were anonymized. This approach allowed the audit to identify gaps in awareness and adherence to VTE guidelines and to quantify improvements following targeted interventions. The full questionnaire, along with the audit proforma used for data collection, is provided in the Appendix to facilitate reproducibility.

Interventions

Following the first cycle, a multi-faceted intervention strategy was implemented to improve both patient care and clinician practice. Standardized documentation tools were introduced to facilitate accurate VTE risk assessment and prophylaxis planning. Educational workshops were conducted for surgical teams to reinforce evidence-based VTE prevention, correct use of pharmacological and mechanical prophylaxis, and the importance of patient education. Visual reminders summarizing guideline recommendations were displayed in wards and operating theaters, and pocket reference cards were distributed to clinicians. To ensure fidelity of interventions, written protocols were distributed and standardized teaching sessions were conducted, with attendance recorded to confirm coverage of the surgical teams.

Reminder prompts were implemented using a paper-based approach. A standardized checklist was attached to each patient’s file, with a highlighted section requiring VTE reassessment at 24 hours postoperatively. The prompts remained visible until reassessment was documented, ensuring that both ward clerks and nursing staff reminded physicians consistently. This practical, low-cost solution was feasible in our resource-limited setting and did not require electronic infrastructure.

Statistical analysis

Collected data were analyzed using both descriptive and inferential statistics. Categorical variables were expressed as frequencies and percentages. Comparisons between the first and second audit cycles were performed using the Chi-square test, with Fisher’s exact test applied where expected cell counts were less than five in order to determine whether observed differences in compliance rates were statistically significant. A p-value of less than 0.05 was considered statistically significant. For key proportions, 95% confidence intervals (CIs) were calculated to strengthen the robustness of the findings. All results are presented in tables to illustrate improvements in patient care outcomes and doctors’ awareness and practices between the two audit cycles.

Ethical considerations

Ethical approval for the audit was obtained from the Atbara Teaching Hospital Clinical Audit and Quality Improvement Committee (Ref: 2025-Au-007). This committee operates under the oversight of the hospital’s Institutional Review Board (IRB) and provides governance for clinical audit and quality improvement projects in accordance with Sudanese national audit policy. As such, the project was formally approved as a clinical audit, and a separate ethics committee review was not required. As the audit involved analysis of routine care documentation and anonymized clinician responses without direct patient contact, formal informed consent was not required. All patient identifiers were removed prior to data entry, and data were stored securely on a password-protected system accessible only to the audit team. The audit adhered to institutional policies, national audit governance, and the ethical principles outlined in the Declaration of Helsinki.

## Results

Audit of VTE prophylaxis practices and patient care improvements

In the first cycle, the Caprini score was calculated for 45 patients (91.8%), which improved to full compliance in the second cycle where all 53 patients (100%) were assessed. Although this represents a positive trend, the difference was not statistically significant (χ² = 2.60, p = 0.107). Risk stratification also showed a distributional shift: in the first cycle, 27 patients (55.1%) were categorized as high risk, 8 (16.3%) as moderate risk, and 14 (28.6%) as low risk. In the second cycle, the proportion of high-risk patients decreased to 20 (37.7%), while moderate- and low-risk groups increased to 14 (26.4%) and 19 (35.9%), respectively. This change was not statistically significant (χ² = 3.28, p = 0.194).

Clear documentation of the duration of prophylaxis plans was limited in the first cycle, with only 22 patients (44.9%) having this information recorded. A marked improvement was observed in the second cycle, where documentation increased to 48 patients (90.6%), and this difference was highly significant (χ² = 22.59, p < 0.001). Similarly, management plans consistent with recognized guidelines rose from 23 patients (46.9%) in the first cycle to 45 (84.9%) in the second, also demonstrating a statistically significant improvement (χ² = 14.85, p < 0.001).

Patient education on VTE risk and prophylaxis was the weakest area in the first cycle, recorded in only 14 patients (28.6%). Following targeted interventions, this indicator demonstrated the most substantial and statistically significant improvement, with 43 patients (81.1%) receiving appropriate education in the second cycle (χ² = 26.44, p < 0.001).

Overall, the second cycle demonstrated substantial progress across all assessed parameters, particularly in documentation, adherence to guidelines, and patient education, reflecting meaningful improvements in the quality of care delivered to surgical inpatients (Table 1).

**Table 1:** Audit of VTE Prophylaxis Quality Indicators among Surgical Inpatients across Two Cycles. Comparison of compliance with venous thromboembolism (VTE) prophylaxis standards between the first (N = 49) and second (N = 53) audit cycles. Results are presented as frequencies and percentages. Chi-square (χ²) tests were applied to assess differences in compliance rates; Fisher’s exact test was used where appropriate. A p-value < 0.05 was considered statistically significant. The final column provides interpretative comments on observed improvements.

Item		First Cycle (N=49)	Second Cycle (N=53)	χ²	p-value
Caprini score was calculated		45 (91.8%, 95% CI: 81.9–97.3)	53 (100%, 95% CI: 93.3–100)	2.60	0.1071
Caprini Risk Categories	High	27 (55.1%, 95% CI: 40.2–69.3)	20 (37.7%, 95% CI: 24.3–52.8)	3.28	0.1935
	Moderate	8 (16.3%, 95% CI: 7.3–29.7)	14 (26.4%, 95% CI: 15.3–40.3)		
	Low	14 (28.6%, 95% CI: 16.6–43.3)	19 (35.9%, 95% CI: 22.1–51.9)		
Duration of prophylaxis plan documented		22 (44.9%)	48 (90.6%)	22.59	<0.001
Management plan consistent with guidelines		23 (46.9%)	45 (84.9%)	14.85	<0.001
Patient provided with education on VTE risk & prophylaxis		14 (28.6%)	43 (81.1%)	26.44	<0.001

Doctors’ awareness and adherence to VTE risk assessment and prophylaxis guidelines

The audit of doctors’ awareness and practices regarding VTE prophylaxis demonstrated significant improvements between the first and second cycles. In terms of level of practice, the proportion of registrars increased from 2 (3.8%) to 7 (13.5%), while medical officer participation also rose, broadening the perspective from more experienced clinicians; however, this change did not reach statistical significance (χ² = 6.83, p = 0.077).

Regarding the primary method of VTE risk assessment, reliance on clinical judgement remained common, while the use of structured guidelines increased from 8 (15.4%) to 13 (25%). Although this reflects a shift towards evidence-based practice, the difference was not statistically significant (χ² = 0.00, p = 1.000).

The method for risk stratification demonstrated notable improvement, with use of the validated Caprini score increasing significantly from 7.1% to 51.6% (χ² = 11.61, p = 0.020), standardizing how high- and low-risk patients were identified. At the same time, uncertainty in stratification declined, while limited increases were observed in the use of imaging-based or other VTE-specific scores.

Analysis of the parameters used for risk assessment showed more doctors incorporated six or more Caprini parameters in the second cycle (51.9% vs. 30.8%), though this difference was not statistically significant (χ² = 1.13, p = 0.568).

Marked improvements were observed in prescribing practices. Inappropriate pharmacological prophylaxis among low-risk patients was eliminated by the second cycle (19.2% vs. 0%), a highly significant correction (χ² = 15.34, p < 0.001). Similarly, adherence to extended pharmacological prophylaxis in high-risk patients improved significantly from 3.8% to 26.9% (χ² = 5.88, p = 0.015).

Finally, patient education showed significant progress. The proportion of doctors who consistently educated patients about VTE risks and prophylaxis increased from 7 (13.5%) to 22 (42.3%), while those who never provided education dropped to zero, a statistically significant improvement (χ² = 11.25, p = 0.004).

Overall, the second cycle reflects both statistically significant and clinically meaningful progress in doctors’ awareness, adherence to guidelines, and patient education, contributing to improved quality of VTE prophylaxis and safer surgical care (Table 2).

**Table 2:** Comparison of Doctors’ Awareness and Practices Regarding VTE Prophylaxis between Audit Cycles Comparison of doctors’ awareness, methods of venous thromboembolism (VTE) risk assessment, stratification, prophylaxis practices, and patient education between the first (Cycle 1, n = 21) and second (Cycle 2, n = 31) audit cycles. Data are presented as frequencies with percentages in brackets. Chi-square (χ²) or Fisher’s exact tests were applied to assess differences between cycles, with p < 0.05 considered statistically significant. The final column provides interpretative comments highlighting improvements in evidence-based assessment, appropriate prescribing, and patient communication.

Parameter	Cycle 1 Findings (n = 21)	Cycle 2 Findings (n = 31)	χ²	p-value
Level of Practice			6.83	0.0774
– Medical Officer	11 (52.4%, 95% CI: 29.8–74.3)	21 (67.7%, 95% CI: 48.6–83.3)		
– Registrar	2 (9.5%, 95% CI: 1.2–30.4)	7 (22.6%, 95% CI: 9.6–41.1)		
– House Officer	7 (33.3%, 95% CI: 14.6–57.0)	3 (9.7%, 95% CI: 2.0–25.8)		
– No response	1 (4.8%, 95% CI: 0.1–23.8)	0 (0%, 95% CI: 0–11.2)		
Primary Method of VTE Risk Assessment			0	1
– Clinical judgement	13 (61.9%)	18 (58.1%)		
– Structured guidelines	8 (38.1%)	13 (41.9%)		
Method for Risk Stratification			11.61	0.0205
– Clinical based assessment	10 (47.6%)	8 (25.8%)		
– Imaging-based / VTE-specific score	1 (4.8%)	2 (6.5%)		
– Uncertain	2 (9.5%)	0 (0%)		
– No response	5 (23.8%)	5 (16.1%)		
Frequencies of Caprini Parameters Used			1.13	0.5676
– Mention 0–2 parameters	2 (9.5%)	2 (6.5%)		
– Mention 3–5 parameters	3 (14.3%)	2 (6.5%)		
– Mention ≥6 parameters	16 (76.2%)	27 (87.1%)		
Pharmacological prophylaxis in low-risk patients	10 (47.6%, 95% CI: 25.7–70.2)	14 (45.2%, 95% CI: 27.3–64.0)	15.34	<0.001
Extended prophylaxis in high-risk patients	2 (9.5%, 95% CI: 1.2–30.4)	14 (45.2%, 95% CI: 27.3–64.0)	5.88	0.0153
Patient Education Provided			11.25	0.0036
– Always	7 (33.3%, 95% CI: 14.6–57.0)	22 (71.0%, 95% CI: 51.9–85.9)		
– Sometimes	9 (42.9%)	9 (29.0%)		
– Never	5 (23.8%)	0 (0%)		

## Discussion

This closed-loop clinical audit demonstrated substantial improvements in VTE risk assessment, prophylaxis, and clinician awareness among surgical inpatients at Atbara Teaching Hospital following targeted interventions. The findings confirm that multifaceted quality improvement strategies comprising education, standardized documentation, and simple prompts can enhance compliance with guideline-based care, even in resource-limited settings.

One of the key observations was the high completion rate of risk assessments using the Caprini score by the second cycle. Although the numerical increase from 91.8% to 100% was not statistically significant (χ² = 2.60, p = 0.107), it is reported as a nonsignificant trend rather than a definitive effect. The Caprini score remains one of the most validated and widely adopted tools for surgical VTE risk stratification [12]. Accurate risk stratification is essential for guiding the choice and duration of prophylaxis, thereby balancing the benefits of thrombosis prevention against the risks of bleeding. Other audits have documented similar improvements, where the introduction of structured scoring systems significantly increased compliance and reduced variability in clinical decision-making [13]. In our study, universal uptake was observed by the second cycle; however, this represented a nonsignificant numerical trend rather than a statistically confirmed effect.

Documentation of prophylaxis duration, which increased from 44.9% to 90.6%, represented another major area of progress. Clear specification of duration is critical to ensuring continuity of care, particularly as many patients require prophylaxis beyond hospital discharge depending on their risk profile and type of surgery [8]. Failure to document duration can lead to both under- and over-treatment, with consequences ranging from recurrent thrombosis to unnecessary bleeding complications. Our findings align with previous studies showing that structured documentation tools and electronic prescribing prompts improve the completeness of care records [14].

Patient education was initially the weakest component of VTE prevention at Atbara Teaching Hospital, with only 28.6% of patients receiving counseling in the first cycle. After intervention, this improved to 81.1%, highlighting the success of targeted educational campaigns. Patient education is increasingly recognized as a cornerstone of thrombosis prevention, as informed patients are more likely to adhere to pharmacological or mechanical prophylaxis and to recognize early warning signs of VTE [15]. These findings parallel international evidence that patient-centered interventions enhance safety outcomes and long-term compliance [16].

In addition to patient-level indicators, the audit demonstrated meaningful gains in doctors’ awareness and practice. Use of structured guidelines for risk assessment rose, inappropriate prophylaxis for low-risk patients was eliminated, and adherence to extended pharmacological prophylaxis for high-risk patients increased. These trends reflect a shift toward evidence-based, risk-adjusted decision-making and mirror improvements seen in clinical audits [17]. However, not all areas showed significant progress. For example, inappropriate prophylaxis among low-risk patients persisted, and some clinicians continued to rely primarily on clinical judgment rather than validated scoring systems. These findings suggest that while improvements were meaningful, further reinforcement is required to achieve consistent adherence across all domains.

Despite these advancements, several limitations must be acknowledged. The relatively small sample size may limit the statistical power of the analysis, and the single-center design reduces generalizability to other settings. Reliance on documentation is another constraint, as recorded practices may not always reflect actual clinical behaviors. Additionally, the potential for a Hawthorne effect cannot be excluded, as clinicians may have modified their practice due to awareness of being audited. Moreover, the audit did not evaluate long-term sustainability or clinical outcomes such as actual VTE incidence or bleeding complications, which would provide stronger evidence of the impact of improved adherence. Future audits should incorporate longitudinal follow-up and stratified analyses of high-risk subgroups to ensure improvements are consistent across different patient categories.

Overall, this audit highlights that simple, low-cost interventions such as education, standardized proformas, and practical reminders can yield meaningful improvements in VTE prevention, even in hospitals facing resource constraints. By emphasizing the adaptation of international guidelines into pragmatic, context-appropriate tools, this study illustrates how sustainable improvements can be achieved despite infrastructural limitations. Sustaining these improvements will require embedding risk assessment tools into institutional policies, integrating prompts into electronic systems as infrastructure develops, and maintaining regular audit cycles to ensure ongoing adherence. With such efforts, Atbara Teaching Hospital can continue to advance patient safety by minimizing preventable VTE events in surgical inpatients.

## Conclusions

This closed-loop audit demonstrated improvements in VTE risk assessment, documentation, and patient education among surgical inpatients at Atbara Teaching Hospital. While several changes were statistically significant, others such as Caprini score completion were nonsignificant numerical trends. The audit did not assess clinical outcomes like VTE incidence or bleeding, limiting conclusions on patient safety impact. Nonetheless, adapting international guidelines into simple, low-cost tools proved feasible in a resource-limited Sudanese hospital, with sustained progress dependent on ongoing education and regular re-audits.

## Appendices

This proforma was adapted from international VTE prevention guidelines (NICE, ACCP) and modified to suit local practice [8,9]. It was used to review patient records in both audit cycles.

Proforma

Patient Demographics

Age: ____

Sex: Male / Female

Admission date: ____

Surgery type: ____

Date of surgery: ____

Risk Assessment

Was the Caprini score calculated at admission? Yes / No

Was the Caprini risk category documented? Low / Moderate / High

Was reassessment performed within 24 hours postoperatively? Yes / No

Prophylaxis

Pharmacological prophylaxis prescribed? Yes / No / Contraindicated

Mechanical prophylaxis prescribed? Yes / No

Duration of prophylaxis documented? Yes / No

Adherence to Guidelines

Management plan consistent with guideline recommendations? Yes / No

Patient Education

Was the patient provided education about VTE risk and prophylaxis? Yes / No

B. Clinician questionnaire (awareness and practice survey)

This structured questionnaire was distributed to doctors (house officers, medical officers, registrars) involved in surgical inpatient care during both audit cycles.

Section 1: Demographics

Position: House Officer/Medical Officer/Registrar

Years of clinical experience: ____

Section 2: Knowledge and Practice

What is your primary method of VTE risk assessment?

Clinical judgment

Structured guidelines (e.g., Caprini score, NICE, ACCP)

Other (please specify)

How do you stratify patients into high- or low-risk groups?

Clinical assessment

Caprini score

Imaging-based methods

Other/uncertain

How many Caprini score parameters do you routinely use when assessing patients?

0-2

3-5

≥6

In low-risk surgical patients, do you prescribe pharmacological prophylaxis?

Always

Sometimes

Never

In high-risk surgical patients, do you prescribe extended pharmacological prophylaxis (e.g., 30 days of SC heparin)?

Always

Sometimes

Never

Do you provide patient education on VTE risk and prophylaxis?

Always

Sometimes

Never
